# Tocilizumab-induced inflammatory flare in Takayasu arteritis: a case report with exploratory mechanistic findings of Fcγ receptor involvement

**DOI:** 10.3389/fimmu.2026.1898851

**Published:** 2026-08-04

**Authors:** Ning Shen, Xuexiao Jin, Panpan Lv, Xiaoping Chen, Xiayan Xu, Linrong Lu, Yongmei Han

**Affiliations:** 1 Department of Rheumatology, Sir Run Run Shaw Hospital, Zhejiang University School of Medicine, Hangzhou, China; 2 Zhejiang Key Laboratory of Biotherapy, Hangzhou, China; 3 Department of Ultrasound, Sir Run Run Shaw Hospital, Zhejiang University School of Medicine, Hangzhou, China; 4 Zhejiang University School of Medicine, Hangzhou, China; 5 Institute of Immunology, Zhejiang University School of Medicine, Hangzhou, China; 6 Shanghai Immune Therapy Institute, Renji Hospital, Shanghai Jiao Tong University School of Medicine, Shanghai, China

**Keywords:** FCR activity, inflammatory flare phenomenon, Takayasu arteritis (TAK), tocilizumab (TCZ), tofacitinib

## Abstract

**Background:**

To report a refractory Takayasu arteritis (TAK) patient who developed a severe inflammatory flare after tocilizumab (TCZ) therapy and explore the underlying mechanism.

**Case presentation:**

A 21-year-old female with active TAK received TCZ after insufficient response to prednisone. TCZ was temporarily withheld then re-added at 400 mg due to disease relapse. After TCZ reintroduction, the patient developed worsening vascular pain, new abdominal pain, dizziness, fever, and markedly elevated inflammatory markers. Imaging showed worsened arteritis and new superior mesenteric arteritis. Methylprednisolone pulse and infliximab failed to control the flare, but tofacitinib achieved complete symptom resolution and normalized inflammatory markers within one month.

***In vitro* findings:**

Peripheral blood samples of the patient were collected and peripheral blood mononuclear cells (PBMCs) Fcγ receptor (FcγR) expression and downstream signaling were assessed upon TCZ stimulation. *In vitro* studies showed that increasing TCZ concentrations were associated with enhanced FcγRIIA, FcγRIIB, and FcγRI expression, along with increased spleen tyrosine kinase (SYK) phosphorylation in the patient’s PBMCs.

**Conclusion:**

TCZ can paradoxically trigger an inflammatory flare in refractory TAK, with exploratory evidence suggesting possible involvement of excessive FcγR activation. Non-antibody-based Janus kinase (JAK) inhibitors like tofacitinib may be effective rescue therapy in such cases.

## Introduction

Takayasu arteritis (TAK) is a chronic, progressive, granulomatous vasculitis predominantly affecting the aorta and its major branches. It mainly affects young women and is associated with increased mortality due to ischemia, aneurysm, and other vascular complications (1). The management of refractory TAK remains a significant challenge.

Elevated serum interleukin-6 (IL-6) levels in TAK patients have been reported, suggesting that IL-6 may serve as a valuable biomarker for disease activity and prognosis (2, 3). This strong association provides a compelling rationale for IL-6 receptor (IL-6R) blockade in the management of TAK. Tocilizumab (TCZ), a humanized monoclonal IgG1 antibody targeting the IL-6R, has been recognized as an effective treatment for TAK, particularly in patients who are refractory to conventional therapies (4, 5).

However, treatment responses to TCZ in TAK are heterogeneous. While many patients benefit, a subset exhibits inadequate response, including progressive arterial inflammation, new-onset vascular lesions, or aortic ulcer formation (6, 7). Even more concerning is the rare but severe macrophage-activation syndrome following TCZ administration (8).

Here, we report a refractory TAK patient who developed a severe inflammatory flare after TCZ reintroduction. We also present exploratory *in vitro* findings suggesting possible Fcγ receptor (FcγR) dysregulation and describe successful rescue therapy with a Janus kinase (JAK) inhibitor.

## Methods

### Case presentation and data collection

A 21-year-old female presented with a 3-month history of back and neck pain, headache, toothache, and fever. Positron emission tomography-computed tomography (PET-CT) confirmed Takayasu arteritis, revealing multiple arteritis involving the left carotid, brachiocephalic trunk, subclavian arteries, aortic arch, and superior mesenteric artery. The patient’s treatment course and disease activity were longitudinally monitored. Clinical symptoms including fever and vascular pain, and laboratory inflammatory parameters including C-reactive protein (CRP) and erythrocyte sedimentation rate (ESR) were recorded at each visit. Ultrasound conducted by the same experienced musculoskeletal and vascular ultrasound specialist, who was blinded to the patient’s detailed clinical condition, was used for vascular assessments at each visit. The maximal intimal-media thickness (IMT) and maximal stenosis rate assessed by ultrasound were used to evaluate the severity of arterial inflammation (9). The Indian Takayasu Clinical Activity Score (ITAS)2010, ITAS.CRP, and ITAS.ESR were used for disease activity evaluation, and active disease was defined as ITAS2010 ≥ 2 and/or ITAS.CRP/ITAS.ESR ≥ 5 (10, 11). Written informed consent was obtained for publication and associated laboratory investigations. The details regarding the disease’s response to TCZ, treatment-related adverse reactions, and treatment adjustments were meticulously documented.

### *In vitro* FcγR expression and signaling assay

Peripheral blood mononuclear cells (PBMCs) were isolated from the patient at four independent time points during stable disease phase, including two time points at approximately six months after the inflammatory flare (on prednisone 15 mg/day and tofacitinib [TOF] 10 mg/day), and two time points at approximately two years after the inflammatory flare (on prednisone 5 mg/day and TOF 10 mg/day). At each time period, a sex- and age-matched healthy control sample was collected and processed in parallel, including one healthy control matched to the two six-month time points, and another healthy control matched to the two two-year time points. In total, PBMC samples from the patient (n = 4) and two healthy controls with each healthy control contributing two samples (n = 4) were used for the *in vitro* experiments. Cells were cultured in RPMI-1640 with 10% fetal bovine serum. TCZ (Actemra^®^, Chugai Pharmaceutical, Tokyo, Japan) was added at increasing concentrations (0, 10, 100, and 200 μg/mL) for 1 hour. Total protein was extracted using RIPA lysis buffer with 1% Phenylmethylsulfonyl fluoride (PMSF) (Beyotime, Shanghai, China) and quantified by BCA kit (Beyotime). Western blot was performed to assess FcγR expression and spleen tyrosine kinase (SYK) phosphorylation, including FcγRI (CD64), FcγRIIA (CD32a), FcγRIIB (CD32b), FcγRIII (CD16), SYK, and pSYK (all antibodies at 1:1000, from Abcam or Cell Signaling Technology).

## Results

### Baseline characteristics

TAK was confirmed based on the patient’s symptoms (fever and vascular pain, including back and neck pain, headache, and toothache) combined with PET-CT findings revealing multiple arteritis involving the left carotid artery, brachiocephalic trunk, subclavian arteries, aortic arch, and superior mesenteric artery. The patient’s pre-treatment CRP and ESR levels were 126.28 mg/L and 85 mm/h, respectively. Other laboratory evaluation revealed anemia, thrombocytosis, and leukocytosis with neutrophilia (see detailed baseline laboratory results in **Table 1**).

**Table 1:** Laboratory results of the patient on admission.

Parameter	The patient	Normal range
Age/gender	21/F	/
Course of disease, Mo	7	/
White blood cells, ×10^9^/L	**10.7**	3.5 - 9.5
Neutrophils, %	**80.5**	40.0 – 75.0
Hemoglobin, g/L	**78**	115 - 150
Platelets, ×10^9^/L	**486**	125 - 350
Alanine transaminase, IU/L	15	7 - 40
Aspartate transaminase, IU/L	16	15 - 40
Creatinine, *µ*mol/L	52	41 - 81
C-reactive protein, mg/L	**126.28**	< 6.0
ESR, mm/h	**85**	0 - 20
ANCA	Negative	Negative
ANA	Negative	Negative

Mo, months; ESR, erythrocyte sedimentation rate; ANCA, anti-neutrophil cytoplasmic antibodies; ANA, antinuclear antibodies.

Bold values indicate laboratory results outside the normal range.

### Clinical courses

**Figure 1:**
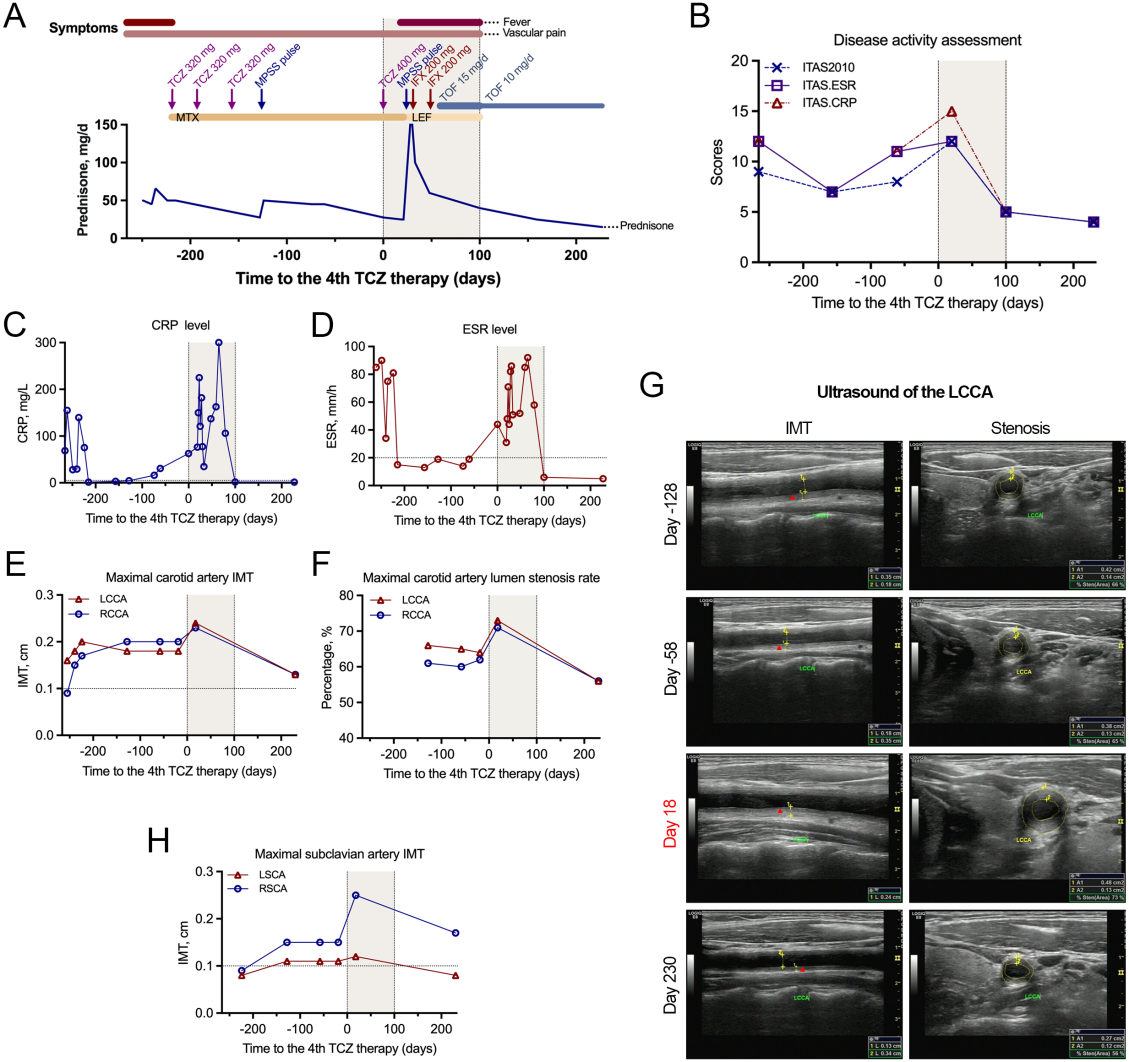
Treatment course and response of the patient. Day 0 = the fourth TCZ treatment; the shaded area (Day 0-100) = inflammation flare following the fourth TCZ treatment. **(A)** Clinical symptoms and treatment attempts. **(B)** Disease activity assessed by the ITAS2010, ITAS.CRP and ITAS.ESR (active disease: ITAS2010 ≥ 2 and/or ITAS.CRP/ESR ≥ 5). **(C)** Trend of CRP. **(D)** Trend of ESR. **(E)** Trend of maximal IMT of bilateral common carotid arteritis. **(F)** Trend of maximal lumen stenosis rate of bilateral common carotid arteritis. **(G)** Ultrasound showing maximal IMT (red triangle) and maximal stenosis rate (% Sten [Area] = [1-A2/A1] * 100) of left common carotid artery. **(H)** Trend of maximal IMT of bilateral subclavian arteritis. TCZ, tocilizumab; ITAS, Indian Takayasu Clinical Activity Score; CRP, C-reactive protein; ESR, erythrocyte sedimentation rate; IMT, intimal-media thickness; LCCA/RCCA, left/right common carotid artery; LSCA/RSCA, left/right subclavian artery.

The patient’s treatment course and disease activity assessment are shown in **Figures 1A, B**. Initial treatment with prednisone at 1 mg/kg for 5 weeks failed to control vascular pain, fever, or elevated CRP (**Figure 1C**) and ESR (**Figure 1D**). As fever and vascular pain persisted despite high-dose steroids, and no immunosuppressants or biologic agents had been used previously, TCZ (320 mg monthly) was added in combination with methotrexate (MTX) based on clinical evaluation and multidisciplinary discussion. Subsequent administration of 3 monthly doses of TCZ (320 mg) normalized the patient’s body temperature, CRP and ESR, but vascular pain persisted. Ultrasound showed even worsened thickening of the right common carotid (**Figures 1E–G**) and bilateral subclavian arteries (**Figure 1H**). Additional pulse therapy with methylprednisolone sodium succinate (MPSS, 500 mg/day for 3 days) was administered, and TCZ was temporarily withheld. Over the following three months, the patient’s vascular ultrasound findings remained stable, but vascular pain was not completely relieved. Given the re-elevated CRP and ESR levels, TCZ was reintroduced at a dose of 400 mg in accordance with the patient’s body weight.

Unexpectedly, the patient developed significant worsening vascular pain within two days, and new-onset of abdominal pain, dizziness, and fever in two weeks. CRP and ESR increased to even higher levels. Vascular ultrasound assessment revealed significantly worsened inflammation and wall thickening in bilateral common carotid and subclavian arteries, as well as newly identified superior mesenteric arteritis on ultrasound. Infectious causes were actively excluded. The patient presented with vascular pain and fever but lacked localizing symptoms of infection, such as cough, sputum, or urinary symptoms. Blood cultures, procalcitonin, chest computed tomography, and SARS-CoV-2 testing were all negative or normal. Screening for tuberculosis and HIV was also negative. Over the following two months, treatment attempts including MPSS pulse (1000 mg/day, 3 days) and infliximab (IFX, 5 mg/kg, two infusions, two weeks apart) failed to curb the inflammation. Adding TOF at 15 mg/day finally controlled the disease. Within one month, this led to the complete resolution of vascular pain and normalized CRP and ESR, enabling successful tapering of TOF to 10 mg/day and prednisone to 25 mg/day. By two years after the flare, the patient remained on prednisone 5 mg/day and TOF 10 mg/day.

### Exploratory *in vitro* mechanistic findings

**Figure 2:**
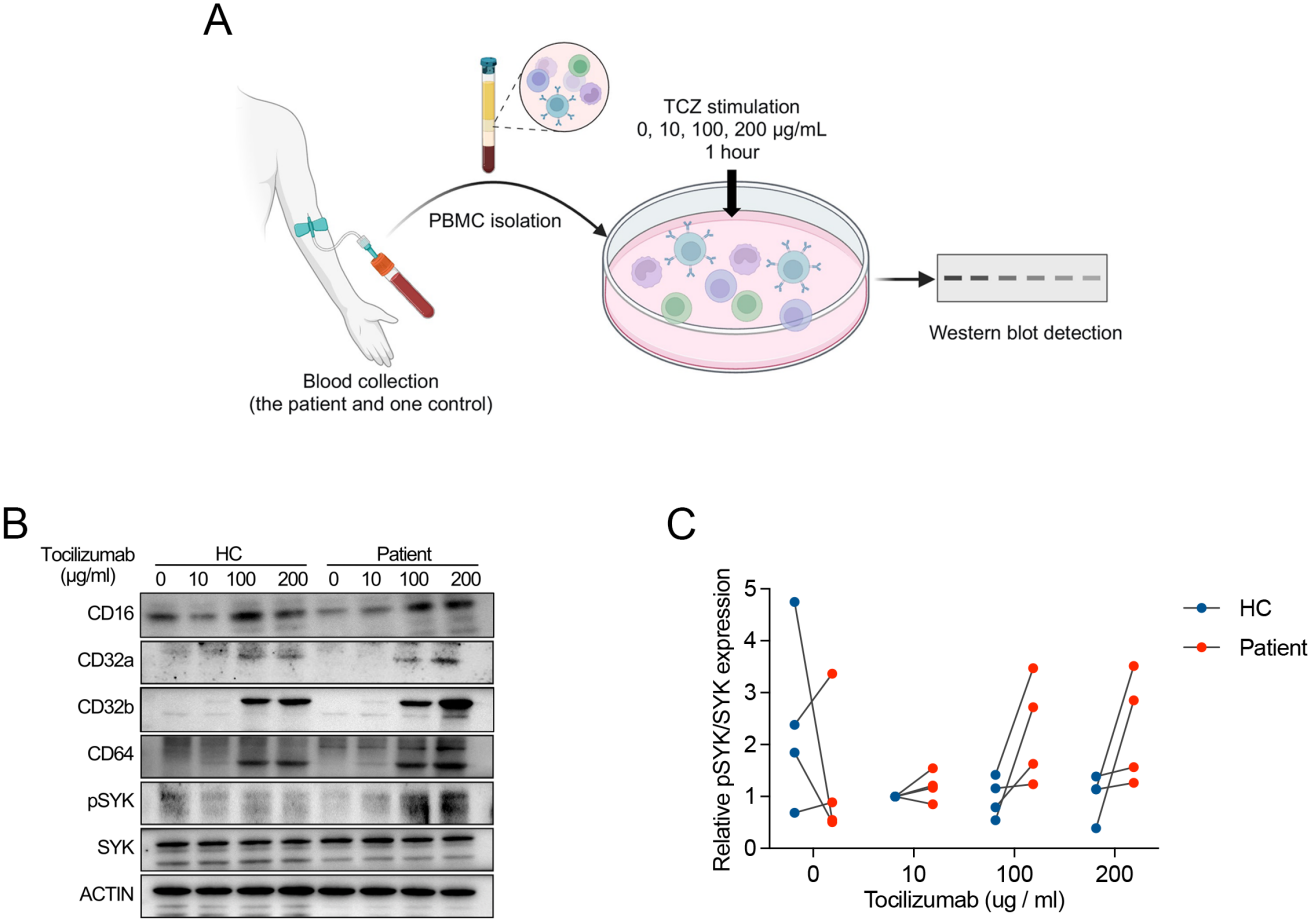
*In vitro* FcγR expression and SYK phosphorylation upon TCZ stimulation. **(A)** Schematic diagram of the experimental procedure. PBMCs from the patient (four independent time points) and two sex-/age-matched healthy controls (one at six-month time point and one at two-year time point post-flare, each sampled twice) were stimulated with increasing concentrations of TCZ (0, 10, 100, and 200 μg/mL). Created using BioRender.com **(B)** FcγR expression and SYK phosphorylation in PBMCs from the patient and a representative healthy control after treatment with different concentrations (0, 10, 100, and 200 μg/mL) of TCZ. The representative blots shown are from samples collected at the first six-month time point. **(C)** Quantitative densitometry of the pSYK/SYK ratio. Data points represent repeated measurements from the patient’s PBMCs (two time points at six months and two at two years post-flare) and matched healthy controls. No statistical inference was drawn from this exploratory analysis. FcγR, Fcγ receptor; SYK, spleen tyrosine kinase; TCZ, tocilizumab; PBMC, peripheral blood mononuclear cell.

To explore the mechanism underlying this paradoxical inflammatory flare, we detected FcγR expression in PBMCs treated with different concentrations of TCZ (**Figure 2A**). At baseline (without TCZ stimulation), no obvious abnormalities in FcγR expression or intracellular signaling were observed in the patient’s PBMCs compared to the healthy control, which did not suggest a constitutive predisposition to FcγR dysregulation in this patient. However, stimulation with increasing concentrations of TCZ was associated with a dose-dependent increase in expression of FcγRIIA (CD32a), FcγRIIB (CD32b), and FcγRI (CD64). FcγRIIB is an inhibitory receptor, suggesting that both activating and inhibitory arms of the FcγR pathway were affected. Notably, we assessed downstream signaling by measuring the phosphorylation of SYK, a critical signaling node downstream of activating FcγRs. Consistent with the changes in receptor expressions, increasing TCZ concentrations were associated with enhanced SYK phosphorylation, raising the possibility of hyperactivation of the FcγR-mediated signaling cascade (**Figure 2B**). Quantitative densitometry revealed a dose-dependent increase in the pSYK/SYK ratio in the patient’s PBMCs compared with the two healthy controls (**Figure 2C**).

These findings suggest that TCZ, as an antibody-based therapeutic, may be capable of engaging and potentially hyperactivating FcγR pathways in susceptible individuals, which might have correlation with excessive inflammatory responses.

## Discussion

In this report, we describe a patient with refractory TAK who developed a paradoxical severe inflammatory flare following the reintroduction of TCZ, and our exploratory *in vitro* findings raise the possibility that FcγR hyperactivation may be involved as a potential underlying mechanism.

In our case, the major conundrum during the initial TCZ therapy was the discordant response between systemic inflammation and local vascular disease. Although three monthly TCZ doses normalized fever, CRP, and ESR, vascular pain persisted and ultrasound showed progressive arterial thickening. This phenomenon has been previously noted in some TAK patients receiving TCZ (12). Three similar TAK patients have been reported with progressive arterial inflammation after TCZ treatment, with one developed aortic ulcer (6, 7). Our case shares the common feature of paradoxical arterial inflammation during TCZ therapy, but differs in several respects. The flare in our patient occurred acutely within days after TCZ rechallenge, whereas the previously reported cases exhibited a more indolent progression. Additionally, we provide exploratory *in vitro* evidence of FcγR/SYK signaling changes, which was not examined in the earlier cases. These differences highlight the heterogeneous nature of TCZ-associated inflammatory responses in TAK and underscore the need for further mechanistic studies. Importantly, suppression of CRP and ESR by IL-6R blockade may not accurately reflect the activity of local vascular lesions. These findings suggest that IL-6 may not be the dominant driver of local vascular inflammation in all patients, or that redundant pro-inflammatory cytokines such as tumor necrosis factor-alpha (TNF-α) may sustain arterial inflammation despite IL-6R blockade. Consequently, even when these systemic markers return to normal, ongoing arterial inflammation cannot be ruled out. This dissociation suggests that reliance on systemic biomarkers alone may be insufficient for guiding treatment decisions, and underscores the importance of incorporating vascular imaging alongside laboratory parameters when evaluating disease activity in TAK.

Unlike the indolent progression seen in some patients, our patient developed an acute, severe inflammation flare after the fourth TCZ dose. Within two days, vascular pain worsened significantly, and within two weeks, new abdominal pain, dizziness, and fever emerged. CRP and ESR rose to levels exceeding pre-treatment values. Ultrasound showed worsening of pre-existing arteritis and new-onset superior mesenteric artery involvement. We consider it more likely that the inflammatory flare was triggered by TCZ rather than representing spontaneous disease progression. Several observations support this interpretation. First, the flare occurred acutely within days of TCZ rechallenge, contrasting with the patient’s prior slow progression, during which she had only vascular pain without fever. Second, TCZ had been withheld for five months before the fourth dose, and corticosteroids were tapered slowly, making withdrawal an unlikely trigger. We also considered anti-drug antibodies, serum sickness-like reactions, and other immune-mediated drug reactions as alternative explanations. Anti-tocilizumab antibodies were not tested, which we acknowledge as a limitation. However, classic serum sickness-like reactions appeared unlikely given the absence of rash or arthralgia, and lack of improvement after drug discontinuation and corticosteroids therapy (13). These findings also argue against other immune-mediated drug reactions. Collectively, the pattern in this patient is distinct from simple lack of efficacy or typical disease flare, resembling instead a hyperinflammatory state potentially related to antibody-mediated immune activation, although definitive exclusion of all alternatives is not possible based on a single case. Switching to the TNF-α receptor antagonist infliximab failed to control the condition. In contrast, a JAK inhibitor successfully managed the inflammation, raising the possibility that antibody-based therapeutic antibodies, rather than the IL-6 receptor blockade alone, may have contributed to the inflammatory flare.

Our *in vitro* experiments offer a potential explanation for the abnormal reaction. We observed dose-dependent upregulated expressions of both activating and inhibitory FcγRs in the patient’s PBMCs, along with enhanced downstream SYK phosphorylation, suggesting activation of FcγR signaling cascade. However, because both activating and inhibitory receptors were upregulated simultaneously, the enhanced SYK phosphorylation should be interpreted as the integrated net output of concurrent activating and inhibitory signals, rather than as evidence of selective pro-inflammatory receptor engagement. FcγRs are widely expressed on the surface of immune cells and mediate phagocytosis, cytotoxicity, and pro-inflammatory mediator release (14). The net functional outcome of FcγR engagement likely depends on the balance between activating and inhibitory signals, as well as additional factors such as receptor density and ligand availability (15). The biological activity of antibody-based therapies, including anti-CD20 and anti-TNF-α, can be regulated by the binding affinity and specificity of human FcγRs (16, 17). Although the mechanisms driving FcγR hyperactivation in this patient remain unclear, we hypothesize that this aberrant response may have involved in the inflammatory flare after TCZ rechallenge. This hypothesis is supported by the clinical observation that IFX, another antibody-based agent, also failed to control the flare, whereas a small-molecule JAK inhibitor that acts independently of FcγRs completely controlled the disease. However, this clinical observation alone does not prove the proposed mechanism. IFX may have failed for other reasons, such as suboptimal TNF-α blockade, treatment timing during a highly active phase, or inadequate response window. Nonetheless, the response to TOF provides clinically valuable information supporting its consideration as a potential rescue therapy in similar challenging conditions.

Several limitations should be considered. First, this is a single case report, and our findings may not be generalizable. In this case, definitive exclusion of all alternatives of the inflammatory flare is not possible, and vascular assessments were primarily based on ultrasound and were not routinely confirmed by cross-sectional imaging. Second, while we demonstrated FcγR upregulation and SYK phosphorylation *in vitro*, we did not identify the root cause of this hyper-responsiveness. Third, we did not measure serum cytokine levels during the inflammatory flare, which could have provided additional insights into the inflammatory cascade. Fourth, functional assays after TCZ stimulation, such as cytokine release, monocyte activation markers, and downstream inflammatory mediators were not performed in this study. Therefore, the functional consequences of the observed FcγR/SYK changes remain to be determined, and our findings should be interpreted as exploratory.

In conclusion, antibody-mediated excessive activation of Fc receptors may underlie atypical reactions to TCZ in a subset of TAK patients. In such scenarios, non-antibody-based agents, such as JAK inhibitors, may be considered in selected refractory cases when clinically appropriate.

## Data Availability

The original contributions presented in the study are included in the article/supplementary material. Further inquiries can be directed to the corresponding authors.
